# Supplementary material for: Prescribing errors in a Brazilian teaching hospital: Causes and underlying factors from the perspective of junior doctors
Source: PLoS One. 2023 Apr 5;18(4):e0284071. doi: 10.1371/journal.pone.0284071 (PMC10075416; doi:10.1371/journal.pone.0284071)
Supplement: S1 File — (ZIP) [file pone.0284071.s001.zip › Co╠üpia de S1_File.pdf]

ROTEIRO DE ENTREVISTA

O objetivo da entrevista é discutir pontos de vista, opiniões e experiências de erros de prescrição. Como você sabe, erros de prescrição são uma causa importante de eventos adversos nos hospitais. Erros ocorrem sempre que as pessoas trabalham sob pressão, mas podem ser reduzidos por meio de estudo sobre erros e suas causas subjacentes.

Nesse estudo estou interessada na segurança dos pacientes, não estou aqui conversando com você porque acho que você cometeu erros, estou aqui por causa do conhecimento profissional e experiência que você possui.

A confidencialidade será assegurada em todos os momentos e as informações analisadas ou relatadas a partir dessa entrevista não permitirão que ninguém o reconheça. Informações sobre o paciente não serão necessárias; no entanto se os pacientes forem mencionados durante a entrevista, seus detalhes serão imediatamente removidos de todos os registros.

Gostaria que você respondesse cada pergunta de maneira franca. Não há respostas certas ou erradas.

Como isso lhe parece? Você tem comentários ou impressões iniciais antes de começarmos a entrevista?

Acabei de iniciar essas entrevistas e seria realmente útil se você puder me dizer se as perguntas não estão muito claras ou podem ser melhor formuladas.

A entrevista será gravada a menos que você se oponha a isso. As gravações serão mantidas em segurança durante a transcrição e depois destruídas/desgravadas.

A entrevista vai durar cerca de 30 minutos.

Você está pronto para começar?

Antecedentes

Você pode me contar um pouco sobre você?

- Local da sua formação médica
- Qual ano de Residência
- Qual a Especialidade
- Quanto tempo no posto
- Treinamento e experiência de prescrição anterior (na escola de Medicina, especialmente no último ano e no hospital)
- Como isso foi ensinado (aulas, tutorias, internato, etc.)
- Avaliações

1ª Parte – Erros de prescrição

Gostaria de falar sobre erros de prescrição usando essa definição; (Entrega do cartão com a definição abaixo)

**Definição de erro de prescrição:**
Um erro de prescrição ocorre quando, como resultado de uma tomada de decisão de prescrição ou escrita/elaboração/digitação da prescrição, há uma redução não intencional significativa na eficácia do tratamento ou aumento no risco de danos ao paciente.

Você tem algum comentário ou pensamento sobre essa definição?

Pela definição vemos que erros podem ocorrer tanto no processo de tomada de decisão quanto no processo de redação da prescrição, e eu gostaria de falar com você

sobre ambos os tipos. Aqui estão alguns exemplos de erros em cada categoria (entregue o cartão com a tabela).

EXEMPLO DE ERROS DE PRESCRIÇÃO

**Elaboração/ escrita da prescrição**	**Tomada de decisão**
Paciente errado	Duração incorreta
Medicamento omitido	Medicamento omitido
Abreviação inapropriada	Horário incorreto
Ilegível	Frequência incorreta
Prescrição incompleta	Via de administração incorreta
Falta de instruções para uso	Dose incorreta
Falta da assinatura do prescritor	Formulação incorreta
Medicamento incorreto	Medicamento prescrito sem indicação<br>Medicamento contraindicado<br>Interação medicamentosa significante<br>Duplicação da terapia<br>Paciente alérgico ao medicamento prescrito

Você poderia pensar agora em uma situação em que você soube que cometeu um erro de prescrição ou uma situação em que você detectou erros de prescrição cometidos por outros médicos residentes. Você poderia me falar sobre isso?

Os erros podem ser vistos por você de "pequenas bobagens" até erros mais sérios. Estou interessada em todos.

Estímulos serão usados para obter informações mais detalhadas sobre o erro em particular:

Você poderia dizer algo mais sobre isso?

Você pode dar uma descrição mais detalhada do que aconteceu?

Áreas a serem cobertas

A natureza do erro

O tipo de erro cometido

Erros de dosagem; Erros de frequência; Erros na escolha do medicamento, como contraindicações, interações, falta de indicação; Erros farmacêuticos, omissão de informações etc.

A medicação envolvida

Dose / frequência / formulação

A condição a ser tratada

Condição do paciente

Gravidade

O erro atingiu paciente?

Em caso afirmativo, quais foram as consequências?

Se não, como você descobriu o erro?

A situação do erro

Quando? Recente?

Hora do dia?

Como você estava se sentindo no momento - cansado, etc., se com pressa, por quê?

Quem mais estava lá na época?

Tipo de ala/setor?

Quanto tempo trabalhou na ala/setor?

Supervisão

Carga de trabalho geral

Você pode descrever o paciente envolvido? Por favor, não mencione nenhum nome/ Idade / personalidade / classe social / etnia

Relação médico - paciente / paciente visto antes / próprio paciente (era seu paciente?) /atendeu a um chamado

Razões para cometer o erro

Falta de apoio, falta de conhecimento, falta de comunicação, falta de informação, lapso ou falha na memória.

Atitude em relação ao erro

Isso aconteceu antes?

Isso tem acontecido desde então?

Isso já aconteceu com mais alguém?

Você acha que havia algo que poderia ter impedido o erro?

O que o superior/chefe ou colegas pensaram sobre a situação?

Como o erro fez você se sentir?

Por quê?/por quanto tempo?

Mudou a maneira como você prescreve agora?

Lidando com o erro

Mecanismos e meios para lidar com prescrição complexa / difícil

Estratégias de enfrentamento empregadas.

2ª Parte: **Experiências e atitudes em relação à educação médica básica e erros**

Como você se sente em relação ao ensino/treinamento que você recebeu durante o curso Medicina?

Se pobre/ruim - por quê?

Se bom - por quê?

Do que você gostaria mais?

Do que você gostaria menos?

Como você gostaria que fosse ensinado?

Como foi a transição entre o aluno e Residente?

Parte de encerramento

Existe alguma outra coisa sobre a qual você gostaria de falar? Ou qualquer coisa que você gostaria de voltar a comentar?

Desligue o gravador

Pós entrevista

Uma carta de agradecimento deve ser entregue para o participante.

Gostaria de agradecer o seu tempo. Essa entrevista é extremamente valiosa para a pesquisa. Se desejar uma cópia da transcrição da entrevista, ela será providenciada. Quando o estudo for concluído, um resumo das descobertas será enviado a você, se

desejar. Enquanto isso, não hesite em contatar-me se tiver dúvidas ou outras questões que gostaria de discutir.

* **These "Interview schedule" was an adaptation of the one used in Lewis et al. [26], with due authorization.**
