# Supplementary material for: Prescribing errors in a Brazilian teaching hospital: Causes and underlying factors from the perspective of junior doctors
Source: PLoS One. 2023 Apr 5;18(4):e0284071. doi: 10.1371/journal.pone.0284071 (PMC10075416; doi:10.1371/journal.pone.0284071)
Supplement: S2 File — (ZIP) [file pone.0284071.s002.zip › Co╠üpia de S2_File.pdf]

Interview topic guide

The goal of the interview is to discuss views, opinions, and experiences of prescribing errors. As you know, prescription errors are a major cause of adverse events in hospitals. Errors occur whenever people work under pressure, but they can be reduced through the study of errors and their underlying causes. In this study I'm interested in patient safety, I'm not speaking to you because I think you have made mistakes, but because of the professional knowledge and experience you possess.

Confidentiality is assured at all times, and the information analysed or reported from this interview will not allow anyone to recognise you. Patient information is not required; however if patients are mentioned during the interview, their details will be immediately removed from all records.

I would like you to answer each question in a straightforward manner. There are no right or wrong answers.

How does this sound to you? Do you have any comments or impressions before we start the interview?

These interviews have just started and it would be helpful if you could inform me if the questions are not very clear or could be better worded.

The interview will be recorded unless you object to this. The recordings will be kept securely during transcription and then destroyed/unrecorded.

The interview will take about 30 minutes.

Are you ready to begin?

Background

Can you tell me a little about yourself?

- Place of medical education?

- What year of residency?

- Specialty?

- How long in post?

- Previous prescribing training and experience (at medical school, especially in the final year, and in hospital)

- How this was taught (lectures, tutorials, internship, etc.)?

- Assessments?

Part 1 – The prescribing errors

I would like to talk about prescribing errors using this definition; (Hand out card with definition below)

**Definition of prescribing errors:**
A prescribing error occurs "when, as a result of a prescribing decision or prescription writing process, there is an unintentional, significant reduction in the probability of treatment being timely and effective or increase in the risk of harm when compared with generally accepted practice".

Do you have any comments or thoughts regarding this definition?

From the definition we see that errors can occur in both the decision making process and the prescription writing process. I'd like to talk to you about both types. Here are some examples of errors in each category (hand out the card with the chart).

Example of prescribing errors

**Preparing/writing the prescription**	**Decision making**
Wrong patient	Incorrect duration

Omitted medication	Omitted medication
Improper abbreviation	Wrong time
Illegible	Incorrect frequency
Incomplete prescription	Incorrect administration route
Lack of instructions for use	Incorrect dosage
Lack of prescriber's signature	Incorrect formulation
Incorrect medication	Medication prescribed without indication<br>Contraindicated drug<br>Significant drug interaction<br>Therapy duplication<br>Patient allergic to prescribed drug

Now, can you think of a situation where you knew you made a prescribing error or a situation where you detected errors in prescriptions made by other junior doctors. Can you tell me about it?

Errors can be anything, from "minor silly things" to more serious errors. I am interested in all.

Prompts will be used to obtain more in-depth information regarding the particular error:

Could you say something more about that?

Can you give me a more detailed description of what happened?

<u>Areas to be covered</u>

The nature of the error

The type of error made

Dosing errors; frequency errors; errors in choice of drug such as contraindications, interactions, lack of indication; pharmaceutical errors, omission of information, etc.

The medication involved

Dose / frequency / formulation

The condition being treated

Commonness of condition

Severity

Did the error reach the patient?

If so what were the consequences?

If not, how did you find out about the error?

The situation of the error

When? Recent?

Time of day?

How were you feeling at the time – tired? if in a rush then why?

Who else was there at the time?

Type of ward?

How long worked on ward?

Supervision?

General workload?

Can you describe the patient involved? Please do not mention any names.

Age/personality

Doctor-patient relationship/seen patient before/ own patient/on-call

Reasons for making the error

Lack of support, lack of knowledge, lack of communication, lack of information, a lapse or slip in memory.

Attitude towards the error

Has this happened before?

Has this happened since?

Has this happened to anyone else?

Do you think there was anything that could have prevented the error?

What did the consultant or colleagues think about the situation? How did the error make you feel?

Why? /how long for?

Did it change the way you prescribe?

Coping with the error

Mechanisms and means of coping with complex/difficult prescribing

Coping strategies employed

Part 2: Experiences and attitudes towards basic medical education and errors.

How do you feel about the training/teaching that you received at medical school?

If poor – why?

If good – why?

What would you want more of?

What would you want less of?

How would you like it taught?

How was the transition between student and Resident doctor?

Concluding part

Is there anything else you would like to talk about? Or anything you would like to go back to?

Switch off the tape recorder

Post-interview

A thank you letter should be given to the participant.

I would like to thank you for your time. This interview is extremely valuable to the research. If desired a copy of the interview transcript can be provided. When the study is completed, a summary of the findings will be sent to you if you wish. In the meantime, please feel free to contact me if you have questions or other issues you would like to discuss.

* **These "Interview schedule" was an adaptation of the one used in Lewis et al. [26], with due authorization.**
