# Supplementary material for: Prescribing errors in a Brazilian teaching hospital: Causes and underlying factors from the perspective of junior doctors
Source: PLoS One. 2023 Apr 5;18(4):e0284071. doi: 10.1371/journal.pone.0284071 (PMC10075416; doi:10.1371/journal.pone.0284071)
Supplement: Attachment — Submitted filename: Response to Reviewers.docx [file pone.0284071.s003.docx]

**Response to Reviewers**

**Dear Editor and Reviewers,**

**We appreciate the suggestions.**

**We believe we’ve addressed all of them and that it has greatly improved our manuscript.**

**Thank you!**

Journal Requirements

1. Please ensure that your manuscript meets PLOS ONE's style requirements, including those for file naming.

**Response: Yes, we have reviewed our manuscript and believe it conforms to the PLOS ONE templates.**

**If any details are still in disagreement please allow us to correct them.**

2: Please provide additional details regarding participant consent. In the ethics statement in the Methods and online submission information, please ensure that you have specified what type you obtained (for instance, written or verbal, and if verbal, how it was documented and witnessed).

**Response: We agree with the suggestions and added: “All participants gave their written informed consent before participating. Ethical risks were minimized by adopting total confidentiality regarding the data and identity of the participants (anonymity through impersonal coding), and through rigorous care when explaining and obtaining participants’ consent”. (lines 175 to 178)**

In addition, in the ethics statement in the manuscript Methods, please specify whether participation in the study was voluntary, and whether participants could opt out at any time. Please also state whether participation was encouraged by any particular incentives, and any steps that were taken to ensure that participants did not feel pressurized to participate. Finally, please state whether the ethics committee approved all the recruitment and consent procedures.

**Response: We agree with the suggestions and added: “The study participant was informed through the written consent form and the interviewer that their participation was voluntary, had no financial expense or gain and that they were free to opt out at any time, without any harm or coercion. The interviews were conducted in a private place, out of working hours and the audio was recorded. After being transcribed the audio files were deleted (lines 178 a 182)**

**“The study, recruitment and consent were approved without restrictions by the Human Research Ethics Committee of the Federal University of Uberlandia on 03/28/2018, under process number 2.570.103.**

**All of this information was also in the consent form. See below the model used.**

**FREE AND INFORMED CONSENT FORM**

You are being invited to participate in the research titled " **Prescribing errors from the perspective of junior doctors**", under the supervision of researchers **Prof. Dr. Carlos Henrique Martins** da Silva and **Gislaine Ferraresi Bonella**, who is also a pharmacist at HC-UFU.

In this research we are seeking the perspective (views, beliefs, and experience) of physicians on the possible causes and potential consequences of prescribing errors.

The Free and Informed Consent Form will be collected by the researcher Gislaine. In participating, you will be interviewed about your opinions, recollections, and experiences related to prescribing errors.

With your permission, the interview will be audio recorded. You can request that the recorder be turned off at any point during the interview, which will last between 30 minutes. The interview and the collected data are private and will be kept completely confidential. The audio will be erased after transcription and you will not be identified at any time. Even when publishing the results of the research, your identity will be kept private. The risks refer to confidentiality, secrecy, and privacy. To avoid them, the executing team is committed to complete confidentiality of the data and the identity of the participants (for which we will use anonymity), and to the erasure of the interview after transcription. **No information will be used professionally regarding the participants.**

The immediate benefits will be knowledge, reflection and better understanding of the subject. Other benefits are the development/improvement of organisational protocols and systems to prevent prescribing errors in order to ensure greater physician and patient safety.

You will have no expenses and no financial gain by participating in the research and you are free to opt out at any time without any loss or duress.

You will keep an original copy of this Informed Consent Form.

Any questions regarding the research, you may contact: Prof. Dr. Carlos Henrique Martins da Silva, at the Office of the Medical School, Campus Umuarama, Av. Pará, nº 1720, Uberlândia - MG, CEP: 38405-320; phone: 34-3225-8625. You can also contact CEP - Committee for Ethics in Research with Human Beings at the Federal University of Uberlândia: Av. João Naves de Ávila, nº 2121, bloco A, sala 224, Campus Santa Mônica - Uberlândia -MG, CEP: 38408-100; phone: 34-3239-4131. The CEP is an independent collegiate created to protect the interests of research participants in terms of their integrity and dignity and to contribute to the development of research within ethical standards in accordance with the resolutions of the National Health Council.

Uberlândia, ....... of ……..of 201.......

________________________________________________________

Researchers' signatures

I agree to voluntarily participate in the above mentioned project after having been duly informed.

________________________________

Research Participant

3. Please include a copy of the interview guide used in the study, in both the original language and English, as Supporting Information, or include a citation if it has been published previously.

**Response: We agree with the suggestions and added as supporting information “S1 and S2”.**

Additional Editor Comments :

Dear Authors Please include a section on how the rigor of the data analysis was ensured, and each quotes should have an anonymized details

**Response: We agree with the suggestions and included a section “Rigor of data analysis” (lines 219) and the quotes were referenced using numbers representing the participant (P) code, eg, P 01, P02, etc. (lines 254 to 255)**

[Note: HTML markup is below. Please do not edit.] Reviewers' comments:

**Reviewer #1:** Originality: Analysis of prescribing errors caused by junior doctors have not been adequately evaluated in Brazil, therefore this study is original.

Significance: Determining the burden and characteristics of prescribing errors is fundamental to designing appropriate measures in order to mitigate the risks and harms to the patients, especially a large number of violation errors were detected in this study. I agree that pharmacists’ perceptions during the support in clinical decisions when prescribing can increase the safety of medication uses.

Methodology: There are some suggestions in methodology: (1). This study was conducted in one teaching hospital as compared with seven teaching hospitals as performed by Lewis PJ and Ashcroft DM et al. (2014) in UK, more similar study sites should be recruited to increase the generalizability.

(2). Selection bias was likely to occur due to the no responses from the 632 junior doctors. The low response rate for the recruitment can be reduced when more study sites are employed. Also, surface mail for the invitation may be the other solution.

**Response: We agreed with the suggestion. As we did not find similar studies in Brazil we decided to start the research primarily in a university hospital in order to familiarize ourselves with this type of study and then expand the study to include other university hospitals throughout Brazil to increase generalizability.**

 (3). Independent assessors, both authors G and L were involved in the semi-structured interview that selection bias may occur. Instead, well-trained independent interviewers should be employed. Also, third external independent assessor should be used if there are discrepancies between authors G and L regarding the classifications of different types of prescription errors, unsafe acts and the latent and error-producing conditions.

**Response: We agreed with the suggestions and for further studies we intend to conduct training for the interviewers and add a third external independent assessor.**

Accuracy and quality of results: Well organized table and figures were used for the data presentation. Annotation of the abbreviations of KBM and RBM should be used for Figure 1.

**Response: We have included the annotation of the abbreviations in figure 1.**

Clarity and lucidity of presentation: The authors were able to provide plausible explanation for the results of this study. Since electronic prescription system constituted a remarkable number (n=29) of the error-producing conditions reported by junior doctors, enhancement of the prescription and electronic medical records can be mentioned at the discussion part.

**Response: We agree with the suggestion and added to the discussion a reflection on the need for enhancement of the prescription and electronic medical records (lines 618 to 621). “Considering that, in the present study, the electronic prescribing system was the most highlighted error-producing and latent condition, it is suggested that the design of the electronic prescribing system used may be contributing significantly to the occurrence of errors. Improvements in this system should be considered a priority”.**

Adequacy of references: The references included are adequate. However, some incorrect referencing numbers are found.

**Response: We have agreed and corrected the references.**

**Reviewer #2:** Thank you for inviting me to review this manuscript which describes the prescribing errors in a braziian teaching hospital.

Overall, I find the manuscript somewhat confusing. The authors have used a qualitative approach to explore the causes of medication prescribing errors among junior doctors. However, the results presented are very quantitative. Table 1 and 2. The manuscript is also very wordy. The authors should consider cutting down on the number of words, and make their writing more concise.

**Response: We agree with the suggestions and have improved the text to avoid confusion. We agreed that it is not appropriate in qualitative research to identify percentages and it was not our aim in the study to emphasize such aspects, so we removed them from the text and tables. We also reduced the number of words in the manuscript.**

Introduction I like the model (i.e. Figure 1) which is used to explain the conceptual framework of this study. However, the authors tend to repeat what is in the figure as text. Please avoid this. Conceptual framework should be summarised, and jusitification for selecting this theory should be provided The authors should provide a stronger justification on why this work is needed. Note, that any abbreviations used in the figure (e.g. KBM) should be explained as a footnote. Aim should be changed to explore .... (rather than investigate - which is very quantitative)

**Response: We agree with the suggestions and removed the repetition from the text. We have summarized the conceptual framework (table 1) and justified why we use “Reason’s Accident Causation model” in our study: We “have used “Reason’s Accident Causation model” as it addresses behavioral factors linked to errors, enables integrating individual and systemic approaches, and is widely used in hospital contexts [3,4,17,18, 22-29]”(lines 79 to 81).“The problem of prescription errors seen in an individual manner is associated, in part, with human behavior and its failures. This, in turn, is related to a cognitive and emotional structure not easily understood by quantitative research alone. Hence, we chose a qualitative approach to investigate subjective aspects related to errors and an analysis framework based on Reason’s Accident Causation model” (which approaches an individual and systemic view of errors) (lines 97 to 101). In addition, we have provided a stronger justification for why this study is needed: “In Brazil no qualitative study carrying out an in-depth investigation of the underlying causes of prescribing errors from the perspective of junior doctors has been found. This research is made necessary as it will be useful for clinicians, mainly physicians, nurses, and pharmacists, whose professions are directly linked to medication prescription errors, and also contribute to reducing the risk of harm to patients. Although other international studies have addressed this perspective, the same type of investigation in a different and not yet properly explored environment will, on one hand, contribute to a better global understanding of the problem and, on the other, will connect to problems and the specific socio-cultural reality of developing countries, where interventions are necessary and sought after” (lines 105 to 114). We changed the word investigate to explore.**

Methods Should use more subheadings in this section to aid reading. How was the topic guide developed? should include the questions asked as a table.

Where's the rigour and reflexivity sections?

**Response: We agree with the suggestions and have added more subheadings in the methods section, including a section for:**

**Qualitative approach and research paradigm**

**We conducted a qualitative, descriptive exploratory study, collecting data through individual interviews. Focusing on the subjective and personal dimension of human error, we adopted a constructivist paradigm, assuming that there are multiple and distinct subjective realities (perspectives) [31]. Within this paradigm, we adopted for concept-based exploration of themes, Reason’s Accident Causation model [21]. The epistemic dimension was phenomenological [31], starting from the way reality appears to the participants themselves, taking them as autonomous subjects of medical practice. (lines 119 to 127)**

**Researcher characteristics and reflexivity**

**The author who conducted all the interviews was not known or acquainted with the participants before starting the study. The medical prescriptions are usually printed directly in the pharmacy sector and when a problem is detected, the pharmaceutical team contacts the doctor by phone for clarification. No member of the study taught, supervised, or had any authority over participants in the study.**

**The interviewer was fully aware of her potential assumptions during the interviews, taking care that these did not interfere with the accounts, she tried not to deviate from the questions contained in the interview guide and also tried to ensure that all data could be reviewed and discussed by the entire research team and that disagreements could be discussed and resolved by consensus ensuring neutrality and objectivity to the study. (lines 129 to 140)**

**Context**

**The study was carried out in a teaching hospital and service provider of the Brazilian Unified Public Health System (*Sistema Único de Saúde -* SUS) containing 530 hospital beds and served by a computerized prescription system. This system does not allow the locking of the prescribed medications, but it does have a maximum dose alert for some types of medications, such as high-risk medications.**

**In our study, the term *junior doctor* refers to the doctors who, after finishing their undergraduate degree, go on a residency program to specialize in an area of medicine. The vast majority are newly graduated doctors. (lines 142 to 151)**

**Sampling strategy**

**The sampling universe consisted of doctors from the residency program who were training at the hospital in the period between April and July 2018. First, second and third year residents were included in the study, those with over three years and those on vacation or on sick leave during the study period were excluded.**

**Invitation to participate in the study was initially done through an email to all (632) junior doctors enrolled in the Medical Residency Program, however there were no responses. Thus 40 participants were individually recruited in their workplace, either in person or by telephone. As one of the aims of the study was to identify contextual causes and previous errors in different hospital environments, junior doctors of both genders and from different settings (wards, adult and pediatric intensive care units and emergency care units) were selected. The study also sought to contemplate several medical specialties. None of the recruited participants refused to participate in the study, however, six did not participate due to their impossibility to schedule appointments with the interviewer, which, according to them, was due to their residency schedule and work overload. Theoretical saturation was obtained with 34 interviews [32]. (lines 153 to 169)**

**Ethical issues**

**The study, recruitment and consent were approved without restrictions by the Human Research Ethics Committee of the Federal University of Uberlandia on 03/28/2018, under process number 2.570.103. All participants gave their written informed consent before participating. Ethical risks were minimized by adopting total confidentiality regarding the data and identity of the participants (anonymity through impersonal coding), and through rigorous care when explaining and obtaining participants’ consent. The study participant was informed through the written consent form and the interviewer that their participation was voluntary, had no financial expense or gain and that they were free to opt out at any time, without any harm or coercion. The interviews were conducted in a private place, out of working hours and the audio was recorded. After being transcribed the audio files were deleted. (lines 171 to 182)**

**Data collection, instrument and processing**

**An interview topic guide was used with questions related to the “planning” and “execution” of the prescription (S1 and S2), and was an adaptation of the one used in Lewis et al. [26], with due authorization. This approach provided a detailed account of prescribing decisions (specific behaviors when planning and executing the prescription) [26].**

**The interviews were conducted by the first pharmaceutical author G, as she has over 20 years of experience in hospital pharmacy, routinely dealing with prescription errors, at a time chosen by the participant and respecting his or her availability. The interviews lasted between 15 and 35 minutes and were audio-recorded and transcribed in full.**

**In the interview the participant was asked to report prescribing errors he/she had committed and/or errors detected in prescriptions by other junior doctors. The definition of error used and shared with participants was “when, as a result of a prescribing decision or prescription writing process, there is an unintentional, significant reduction in the probability of treatment being timely and effective or increase in the risk of harm when compared with generally accepted practice” [33]. (lines 184 to 199)**

**Data analysis**

**For a qualitative investigation exploring subjective perspectives we used a Framework Approach [34] for data analysis. A period of familiarization with the raw data (listening to the recordings, reading the transcripts, checking notes with reflective care) was previously undertaken by G and L to ensure that they had relevant information for the study's purpose. Reason’s Accident Causation model [21] was used to categorize and present the data. The development of the analytic framework and a categorization and coding according to “Reason's Accident Causation model” were carefully elaborated by G. This model was the most commonly used theoretical model when considering prescribing errors [3,4,17,18,22-29]. To ensure higher accuracy, the application of this framework and the mapping of the data were performed independently by G and L and the differences were settled by consensus. Tables and organizational charts were constructed to facilitate understanding and interpretation.**

**The computer software program NVivo© [35] was used to assist in the organization of the data. Codes and sub-codes to qualify the 34 cases were previously defined and used during the analysis process to identify potential themes. Prescribing errors were then counted and named according to the taxonomy in Otero-López et al. [36]. (lines 201 to 217)**

**Rigor of data analysis**

**We sought to link the data analysis to theoretical constructs, theories used in preliminary work, and codes derived from the theoretical framework of relevant literature to ensure greater reliability and credibility. National and international recommendations/protocols on good prescription practice and patient safety [37 – 40] were used as objective aspects to guide our interpretation and critical discussion of the data to avoid evaluative bias. To ensure researcher neutrality and objectivity, all data was made available for approval by all the authors, two of whom were senior doctors and experienced researchers in qualitative research (A) and health sciences (C). Once the findings were sufficiently clarified with the chosen structural analysis, the interaction between findings and theories enabled transferability. The relevant findings and interpretations present applicability beyond the greater understanding of the problem, such as encouraging new research (in Brazil and in other countries), promoting critical-reflective evaluation and suggesting improvements in care, benefits for the participants and improvements in patient safety. (lines 219 to 233)**

How was the topic guide developed? should include the questions asked as a table.

**The questions are in the topic guide adapted from the model used in the Lewis et al. [26] study and are now attached as additional files (S1 and S2), so we believe there is no need to include the questions asked as a table in order not to overload the manuscript.**

Results Lines 235-242 should be presented as a table to decrease the number of words.

**Response:** **We accepted the suggestion and created a table (Table 1)**

Table 2: how did the authors collect this data? Did they use a survey form? Very quantitative results For a qualitative paper, I do not see what themes emerged from the participants. I only see a repetition of the conceptual framework (i.e. results are presented according to the conceptual framework). As such, I don't think the authors actually explored why the junior doctors made the prescribing errors.

**Response: Regarding Table 2 (currently it is Table 4), we collected the data through semi-structured questions included in the topic guide (S1 and S2) that referred to this aspect, identifying words and phrases such as:**

**Related to the environment: “Being in a hurry” (time pressure), “running against time” (time pressure), “amount of work” (work overload), “amount of patients”, “lots of patients to see” (work overload), “…many patients to see” (work overload).**

**Related to the team: “there never is one! We always do it by ourselves...” (absent or inadequate tutoring).**

**Related to the patient: “critical patients” (complex patient), “very long prescriptions with many items” (polypharmacy).**

**Related to the task: “if a boss is from one segment and the other from another, or if the shift changes, all practices change” (Inadequate protocol).**

**These are some examples of the participants' speeches that we used as examples in the manuscript and that with the help of the Nvivo software and spreadsheets in excel, made into a table. We have added a note about how this table was made in line 392.**

**In tables 4 (old 2) and 5 (new) (line 445), and in the analysis in general, we made the "themes" clearer. That is, the themes identified and emerged with Reason's theoretical model, were sought, refined and interpreted according to the reports and subjective realities of the participants. Which we believe is relevant and novel, since we did not find similar studies in Brazil and there are important findings that differ from the current literature.**

**Our study followed a pattern widely used in other studies [3,4,17,18,22-29] which allowed comparison and dialogue between the studies.**

Also, the quotes do not have the annonymised details of the junior dr. My general impression is that the authors have not analysed their data in depth sufficiently for a qualitative study.

**Response: Codes were used in our data analysis, however, at first, we did not put the codes in the manuscript to hinder participant identification, however, we agree with the suggestion and added in the selected reports the respective codes. (lines 254 to 255)**

**Response: We accept your suggestions and criticism and have improved the data analysis. We believe we have addressed all suggestions and that it has greatly improved our manuscript. Thank you**

Discussion Too wordy. Needs to be summarised.

**Response: We accept the suggestion**
