# Supplementary material for: Prescribing errors in a Brazilian teaching hospital: Causes and underlying factors from the perspective of junior doctors
Source: PLoS One. 2023 Apr 5;18(4):e0284071. doi: 10.1371/journal.pone.0284071 (PMC10075416; doi:10.1371/journal.pone.0284071)
Supplement: Attachment — Submitted filename: RESPONSE TO REVIEWERS.docx [file pone.0284071.s004.docx]

ANSWER TO REVIEWERS

We thank the referees and the editor for their considerations, which we have treated with care in the responses, review and rewriting of the text.

Reviewer #1:

1) On the style of writing – agree with Reviewer 2 that the writing is lengthy while at the same time not easy to digest the messages that the investigators are trying to put across to readers.

RESPONSE

Thank you for taking part of your time to contribute to the improvement of our manuscript.

We agree that the writing was long and we have cut some unnecessary writings. The subject matter and the reality studied by the research is probably the cause of the message not being easy to digest, however we agree with the reviewers that the text is intelligible and written in standard English.

2) Methodology – despite reading the article, it is not apparent from the account on the timeframe during which the interview with the junior doctors was performed. Was it over a year and the number of errors committed an annual figure?

RESPONSE

The interviews lasted 4 months, from April to July 2018.

The number of errors (105) is not an annual number, as it refers to the entire professional life reported and remembered by the interviewees. We have rewritten the text to make this more prominent.

…in the period between April and July 2018, when the interview was performed.

…throughout their entire professional life.

3)Due to the initial nil response rate on the investigators’ email invitation, they elected to recruited interviewees at their workplace in person or by telephone. Was it a random selection taking into account only the recruitment of doctors from as diverse a background as possible? Otherwise, it could be argued that despite their busy work schedule, only those who have a lot of grievances to air would choose to be interviewed.

RESPONSE

This was a random selection taking into account only the recruitment of junior doctors from a background as diverse as possible.Ex.

"As one of the aims of the study was to identify contextual causes and previous errors in different hospital environments, junior doctors of both genders and from different settings (wards, adult and pediatric intensive care units and emergency care units) were selected. The study also sought to contemplate several medical specialties".

RESPONSE

The 40 doctors approached were identified through a list provided by the residency sector.

We added to the text: "The sector responsible for the medical residency programme made available a list with the junior doctors and their specialties".

4)Was the reporting of errors by recalling from memory by the junior doctors or their peers only? This is subject to recall bias, and the number might not reflect the true magnitude.

RESPONSE

Yes, by memory. Because it is a qualitative research with investigation of the reports of the doctors themselves. This is the usual form of research. The number (105) may not reflect the magnitude of the errors, but the central point of the article is mainly to explore the content of the reports, whatever the magnitude.

We have removed table 2 as its data was not relevant to qualitative research and is likely to confuse the reader. Thank you for the alert.

On the risk of memory bias, it is discussed in the analysis as a threshold for the reader to consider.

5)What about reporting by other healthcare professionals such as nurses and pharmacists?

RESPONSE

They were not the object of this specific research, but have been the subject of others in the country and may be the subject of future research.

6)There were reports of many ‘Violations’ – defined as voluntary actions in which rules are ignored, such as not evaluating the patients before copying and repeating the last prescriptions. While this situation is definitely unsatisfactory, these were forced upon the junior doctors from insufficient allowance of adequate time for them to perform the many duties that they had to perform. Is it appropriate to talk about reprimanding the doctors rather than rectifying the work conditions?

RESPONSE

We focused on the working circumstances as a condition for the occurrence of unsafe acts throughout the text, particularly in the conclusion where we suggest measures to improve these conditions, however, we believe it is appropriate to comment on this other aspect (reprimand) as it is a reality. The focus of the study was not to investigate unsafe acts committed by negligence, imprudence, and legal and ethical aspects of medical practice, which yes, should be treated differently, however, even Reason, and several other authors discuss both aspects. We mentioned reprimanding in the discussion, for the enrichment and reflection of the reader.

7)Many comments from the junior doctors were included in the text. They were, however, shown as disjointed messages. By such presentation to the readers, not only that there seems a risk the comments were taken out of context, sometimes the messages themselves were not entirely clear in meaning. The investigators could just describe a system through which they would categorize these comments and present the categorized results.

RESPONSE

We revised the text and deleted some reports that might seem disconnected or unnecessary. The reports, which are even more numerous than those exposed in this article, were chosen and placed in the text to better illustrate the identified themes and, when analyzing them, we took due care not to decontextualise them. Citing such reports is fundamental and common in qualitative articles. There is a categorization of the results in tables 3 and 4 of the new manuscript

Reviewer #3:

Thanks so much for letting review this revised manuscript. I've taken the time to read your responses to the reviewers as well as the manuscript itself.

Overall, I think the paper still drifts into quantitative presentation when you have used an entirely qualitative approach. I hope these points are swift to address.

RESPONSE

Thank you for taking the time to review and contribute to our manuscript. One point to note about this is that our analysis is a descriptive and phenomenological exploration, and although descriptive, it resembles quantitative research in part. But in fact it is not. However, we agree with your opinion and have removed Tables 2 and 3 as they are not relevant to our results, analysis and discussion.

1) Please clarify how the prescribing errors reported in Table 1 were identified. Were these self reported by practitioners, or were they identified through another aspect of the study.

RESPONSE

Table 1 refers to explanations on the classification and definitions used in Reason's Accident Causation model. The examples we have taken from the reports. We have now added a remark in the description of this table. "The examples were taken from the reports of the participants in this study".

In Figure 1, the prescribing errors/unsafe acts reported were identified from self report, but also with conceptual analysis of these reports through Reason's Accident Causation model.

We added in the description of the Figure: "The active failures/unsafe acts were identified from the self-report of the participants in this research.

2) How were the 40 approached clinicians identified?

RESPONSE

We added to the text: "The sector responsible for the medical residency programme made available a list with the junior doctors and their specialties".

3) Table 2 requires only the demography, and not the wider profile of prescribing errors. These aren't important to the analysis.

RESPONSE

We agreed and removed table 2

4) Table 3 is unnecessary.

RESPONSE

We agreed and removed table 3

5) I suggest you review the COREC checklist for reporting of qualitative research to guide your manuscript. Most of it is done but there are some aspects omitted.

RESPONSE

As it was not indicated which aspects were omitted, we carried out a general review.

Table 1 Consolidated criteria for reporting qualitative studies (COREQ): 32-item checklist

Domain 1: Research team and reflexivity

Personal Characteristics

1. Interviewer/facilitator Which author/s conducted the interview or focus group?

“The interviews were conducted by the first pharmaceutical MD author G, as **she** has over 20 years of experience in hospital pharmacy, routinely dealing with prescription errors,... “

2. Credentials What were the researcher’s credentials? E.g. PhD, MD

Pharmaceutical MD

3. Occupation What was their occupation at the time of the study?

RESPONSE

We add it to the text: The author who conducted all the interviews worked at the hospital pharmacy at the study site but was not known or acquainted with the participants before starting the study.

4. Gender Was the researcher male or female?

RESPONSE

**She** has over 20 years of experience in hospital pharmacy,...

5. Experience and training What experience or training did the researcher have?

RESPONSE

“..she has over 20 years of experience in hospital pharmacy, routinely dealing with prescription errors,...”

Relationship with participants “…was not known or familiar with the participants before starting the study”.

6. Relationship established Was a relationship established prior to study commencement?…

RESPONSE

was not known or familiar with the participants before starting the study.

7. Participant knowledge of the interviewer

What did the participants know about the researcher? e.g. personal goals, reasons for doing the research.

RESPONSE

… was not known or familiar with the participants before starting the study.

We added: Reasons and interests were scientific in order to understand the underlying aspects of prescribing errors and were presented to the participant before starting the interview.

8. Interviewer characteristics What characteristics were reported about the interviewer/facilitator? e.g. Bias, assumptions, reasons and interests in the research topic

RESPONSE

The author who conducted all the interviews worked at the hospital pharmacy at the study site but was not known or acquainted with the participants before starting the study. The medical prescriptions are usually printed directly in the pharmacy sector and when a problem is detected, the pharmaceutical team contacts the doctor by phone for clarification. No member of the study taught, supervised, or had any authority over participants in the study.

The interviewer was fully aware of her potential assumptions during the interviews, taking care that these did not interfere with the accounts, she sought not to deviate from the questions contained in the interview guide, ensured that all data could be reviewed and discussed by the entire research team and that disagreements could be discussed and resolved by consensus ensuring neutrality and objectivity to the study.

We added: Reasons and interests were scientific in order to understand the underlying aspects of prescribing errors.

Domain 2: study design

Theoretical framework

9. Methodological orientation and Theory

What methodological orientation was stated to underpin the study? e.g. grounded theory, discourse analysis, ethnography, phenomenology, content analysis

RESPONSE

We conducted a qualitative, descriptive exploratory study, collecting data through individual interviews. Focusing on the subjective and personal dimension of human error, we adopted a constructivist paradigm, assuming that there are multiple and distinct subjective realities (perspectives) [31]. Within this paradigm, we adopted Reason’s Accident Causation model [21] for the concept-based exploration of themes . The epistemic dimension was phenomenological [31], starting from the way reality appears to the participants themselves, taking them as autonomous subjects of medical practice.

Participant selection

10. Sampling How were participants selected? e.g. purposive, convenience, consecutive, snowball

RESPONSE

Invitation to participate in the study was initially done through an email to all (632) junior doctors enrolled in the Medical Residency Program, however there were no responses. The sector responsible for the medical residency program provided a list of junior doctors and their specialties.

11. Method of approach How were participants approached? e.g. face-to-face, telephone, mail, email

RESPONSE

Thus 40 participants were individually recruited in their workplace, either in person or by telephone. As one of the aims of the study was to identify contextual causes and previous errors in different hospital environments, junior doctors of both genders and from different settings (wards, adult and pediatric intensive care units and emergency care units) were selected. The study also sought to contemplate several medical specialties.

12. Sample size How many participants were in the study?

RESPONSE

40 participants

13. Non-participation How many people refused to participate or dropped out? Reasons?

RESPONSE

None of the recruited participants refused to participate in the study, however, six did not participate due to their impossibility to schedule appointments with the interviewer, which, according to them, was due to their residency schedule and work overload. Theoretical saturation was obtained with 34 interviews [32].

Setting

14. Setting of data collection Where was the data collected? e.g. home, clinic, workplace

RESPONSE

Were individually recruited in their workplace,

15. Presence of non-participants Was anyone else present besides the participants and researchers?

RESPONSE

There was no one else present during the interviews.

“The interviews were conducted in a private place,”

16. Description of sample What are the important characteristics of the sample? e.g. demographic data, date

RESPONSE

The sampling universe consisted of doctors from the residency program who were training at the hospital in the period between April and July 2018 during which the interview was performed. First, second and third-year residents were included in the study, those with over three years and those on vacation or on sick leave during the study period were excluded.

Data collection

17. Interview guide Were questions, prompts, guides provided by the authors? Was it pilot tested?

RESPONSE

An interview topic guide was used with questions related to the “planning” and “execution” of the prescription (S1 and S2), and was an adaptation of the one used in Lewis et al. [26],

18. Repeat interviews Were repeat interviews carried out? If yes, how many?

RESPONSE

No. We added to the manuscript text: "... and the interviews were not repeated and the transcripts were not returned to the participant for comment or correction."

19. Audio/visual recording Did the research use audio or visual recording to collect the data?

RESPONSE

They were audio-recorded

20. Field notes Were field notes made during and/or after the interview or focus group?

RESPONSE

Yes. We added to the manuscript text, "Field notes were taken, however interviews were not repeated and transcripts were not returned to the participant for comment or correction."

21. Duration What was the duration of the interviews or focus group?

RESPONSE

The interviews lasted between 15 and 35 minutes

22. Data saturation Was data saturation discussed?

RESPONSE

Theoretical saturation was obtained with 34 interviews [32].

23. Transcripts returned Were transcripts returned to participants for comment and/or correction?

RESPONSE

No. We added to the manuscript text, "Field notes were taken, however interviews were not repeated and transcripts were not returned to the participant for comment or correction."

Domain 3: analysis and findings

Data analysis

24. Number of data coders How many data coders coded the data?

RESPONSE

The codes and sub-codes were pre-established according to Reason's Accident Causation model. They are detailed in Figure 1.

25. Description of the coding tree Did authors provide a description of the coding tree?

RESPONSE

The Coding tree is represented in Figure 1.

The development of the analytic framework and a categorization and coding according to “Reason's Accident Causation model” were carefully elaborated by G. This model was the most commonly used theoretical model when considering prescribing errors [3,4,17,18,22-29]. To ensure higher accuracy, the application of this framework and the mapping of the data were performed independently by G and L and the differences were settled by consensus. Tables and organizational charts were constructed to facilitate understanding and interpretation.

The computer software program NVivo© [35] was used to assist in the organization of the data. Codes and sub-codes to qualify the 34 cases were previously defined and used during the analysis process to identify potential themes. Prescribing errors were then counted and named according to the taxonomy in Otero-López et al. [36].

26. Derivation of themes Were themes identified in advance or derived from the data?

RESPONSE

Codes and subcodes to qualify the 34 cases were previously defined and used during the analysis process to identify potential themes.

27. Software What software, if applicable, was used to manage the data?

RESPONSE

The computer software program NVivo© [35] was used to assist in the organization of the data.

28. Participant checking Did participants provide feedback on the findings?

RESPONSE

yes

Reporting

29. Quotations presented Were participant quotations presented to illustrate the themes / findings? Was each quotation identified? e.g. participant number

RESPONSE

For a better qualitative understanding of the data, literal reports were selected to illustrate the main results. The quotes were referenced using numbers representing the participant (P) code, eg., P 01, P02, etc.

30. Data and findings consistent Was there consistency between the data presented and the findings? RESPONSE

Yes

31. Clarity of major themes Were major themes clearly presented in the findings?

RESPONSE

The themes were detailed in tables 2 and 3. Those that stood out the most and those that stood out the least were discussed in the text that followed the tables.

32. Clarity of minor themes Is there a description of diverse cases or discussion of minor themes?

RESPONSE

Minor themes were also discussed in the text that followed the tables.

6) An aspect of the qualitative approach is how you as the analysts would assign the prescribing errors in Table 3 to one of Reason's latent factors. e.g. were omissions an RBM, KBM, slip/lapse or violation?

RESPONSE

Omissions (taxonomy of Otero-Lópes et al [36]) could have been caused by either RBM or, KBM, slip/lapse or violation (all of which are unsafe acts). However in our research, **Omissions** were mostly attributed to violations. Example taken from the manuscript:

*“Many violations happened this way. Junior doctors made a* ***copy of the prescriptions, planning to return to the system later to check and update it****, but never did* (that is a violation/unsafe act ("Reason 's Accident Causation model" [21]). *The most common errors related to violations were drugs being unnecessarily prescribed* (Unnecessary medication/table 3 - This is an error type according to the taxonomy of Otero-Lópes et al [36]) *and medication* ***omission*** (Medication Omission table 3 - This is an error type according to the taxonomy of Otero-Lópes et al [36].). *Copying prescriptions from the previous day without properly checking them may perpetuate errors for many days”.*

Note: What is in red does not appear in the text of the manuscript, we just flag it here for your better understanding.

In the results and analysis we did this, for all reports we related **Types of errors** (Otero-Lópes et al [36] taxonomy) with the **Unsafe Acts/Active Failures** (Reason 's model" [21]) and identified the Themes (Table 2- Themes identified from the reports as error-producing conditions and Table 3 - Themes identified from the reports as latent conditions).

**Medication omission, wrong dose, wrong medication, wrong route of administration and wrong patient** were also mostly related to **Slips (unsafe acts)** and had as **error-producing conditions** the **"electronic system"** used at the study site (Table 2 - Themes identified from the reports as error-producing conditions).

See this in the text below, taken from the manuscript, where we analyze this relationship:

*“Most* ***slips*** (“Reason's Accident Causation model”[21]) *were related to* ***medication omission and wrong dose, wrong medication, wrong route of administration and wrong patient*  (**Otero-Lópes et. al. [36])***.*** *Due to name similarity between the drugs and their alphabetical proximity in the prescribing* ***electronic system*** (That is an error-producing condition - Table 2 - Themes identified from the reports as error-producing conditions ), *the interviewee selected the* ***wrong medication*  (**Otero-Lópes et. al. [36])”.

Note: What is in red does not appear in the text of the manuscript, we just flag it here for your better understanding.

In summary, Reason [21] classifies the active failures/unsafe acts as "errors" ( RBM, KBM, slip/lapse) and "violations"; Otero-Lópes et. al. [36] classify the "error types" using a specific taxonomy without any mention to the unsafe act that caused such error. These two analyzed aspects provided support for the identification of the THEMES (Table 2 and 3).

7) I find it interesting that you've decided you're using a phenomenological paradigm, but then use grounded approaches to your data analysis. As I read your manuscript I very much see a well designed thematic analysis approach (particularly as you have used the Framework approach to data management and analysis.)

RESPONSE

The overall approach is phenomenological as it was about understanding the subjectivity of the respondents, but the data management was with the framework and the data analysis approach was thematic. A multi-factorial type of methodology.

8) With regards your analysis, what is your unit of analysis?

RESPONSE

Reason's categorisation of medical errors. Over the course of the research, the central unit was violations as the highlight of the results.

9) As I read your results and analysis I wonder if the analysis has been somewhat superficial. You have made a good descriptive presentation of errors grouped using Reason's approach, but then have considered error-producing conditions almost as a separate entity - this isn't really the right way to consider it (and indeed, Lewis et al. wove these provoking events into the typology of errors to give deep insight into what was going on...) Have a look at your data again and see if there's a relationship between, say, team and environment and RBMs and violations.

RESPONSE

We thank you for your alert for the correction. We corrected the text in order to deepen our analysis and leave the relationship between unsafe acts and their error-producing conditions in greater focus, as it was indeed our intention.

11) Please review some of the language that has permeated into the analysis. Page 22, Line 479 onward, there is very clear potential for attribution bias in some of the direct quotes used, and how that has been interpretted. Demanding that the site of study should be closed down and criminal accusations... may be related to teh passion and hyperbole of the participant but isn't wise to feature in a journal article that your employers will read and want to follow up on. Please consider how these can be paraphrased to retain the relevance to the study outcomes, and not embarass the host site..

RESPONSE

We agree. We modified the text.

Thank you for your considerations and corrections.
