# Supplementary material for: Prescribing errors in a Brazilian teaching hospital: Causes and underlying factors from the perspective of junior doctors
Source: PLoS One. 2023 Apr 5;18(4):e0284071. doi: 10.1371/journal.pone.0284071 (PMC10075416; doi:10.1371/journal.pone.0284071)
Supplement: Attachment — Submitted filename: RESPONSE TO REVIEWER 3.docx [file pone.0284071.s005.docx]

RESPONSE TO REVIEWERS

Reviewer #3: Thanks for taking the time to respond to my comments. I am satisfied with your responses. Well done.

Response: Thank you
